# Intestinal flora: a potential pathogenesis mechanism and treatment strategy for type 1 diabetes mellitus

**DOI:** 10.1080/19490976.2024.2423024

**Published:** 2024-11-09

**Authors:** Shengnan Huang, Fangfang Li, Chunhua Quan, Dan Jin

**Affiliations:** a Immunology Biology Key Laboratory, Yanbian University, Yanji, China; b Department of Immunology and Pathogenic Biology, College of Medicine, Yanbian University, Yanji, China; c Central Laboratory, The Affiliated Hospital of Yanbian University, Yanji, China

**Keywords:** Type 1 diabetes mellitus, intestinal flora, short-chain fatty acids, autoimmune disease

## Abstract

Type 1 diabetes mellitus (T1DM) is a chronic autoimmune disease characterized by destruction of pancreatic β-cells, leading to insulin deficiency and hyperglycemia, and its incidence is increasing year by year. The pathogenesis of T1DM is complex, mainly including genetic and environmental factors. Intestinal flora is the largest microbial community in the human body and plays a very important role in human health and disease. In recent years, more and more studies have shown that intestinal flora and its metabolites, as an environmental factor, regulate the development of T1DM through various mechanisms such as altering the intestinal mucosal barrier, influencing insulin secretion and body immune regulation. Intestinal flora transplantation, probiotic supplementation, and other approaches to modulate the intestinal flora appear to be potential therapeutic approaches for T1DM. This article reviews the dysbiosis of the intestinal flora in T1DM, the potential mechanisms by which the intestinal flora affects T1DM, as well as discusses potential approaches to treating T1DM by intervening in the intestinal flora.

## Introduction

Type 1 diabetes mellitus (T1DM) is a growing health problem around the world, with serious health implications especially in children and adolescents. According to the International Diabetes Federation, the incidence of T1DM is on the rise, with over 1.2 million children and adolescents worldwide diagnosed with T1DM in 2022.^1,2^ Traditionally, T1DM was considered solely an autoimmune disease caused by the destruction of pancreatic β-cells by islet-specific T cells. However, it is now recognized as a multifactorial disorder influenced by genetic factors and environmental triggers, including intestinal flora, viruses, diet, and chemicals.^3^

**Figure 1.**
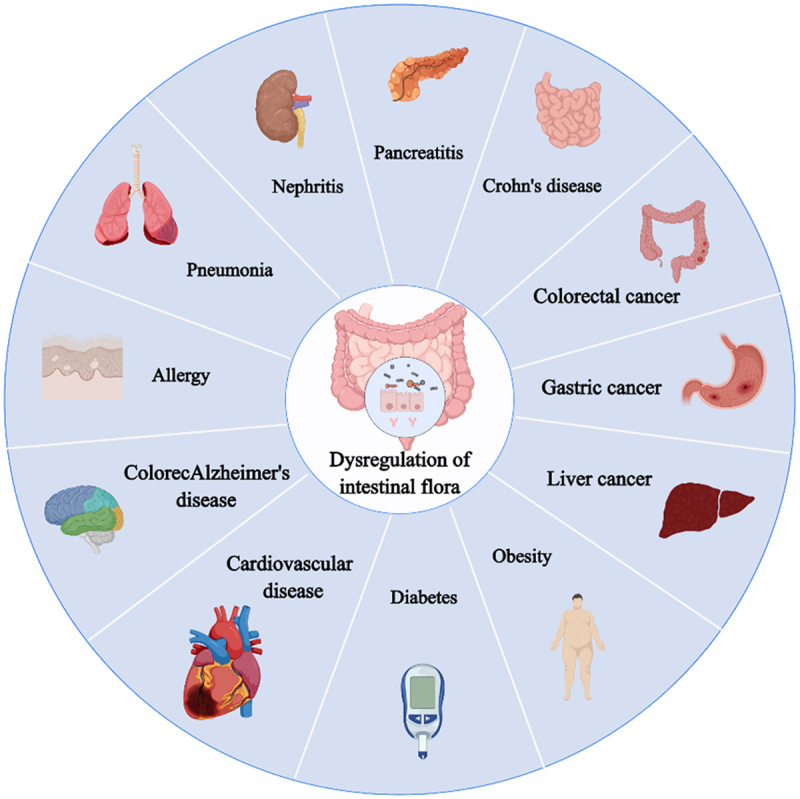
Imbalance of intestinal flora can easily lead to a series of diseases.

The human gut harbors a vast array of microbiota that have co-evolved with the host to form intricate symbiotic relationships. Recent advancements in genomic technologies have provided a deeper understanding of the diversity and composition of intestinal flora.^4^ The intestinal flora and its metabolites not only influence a wide range of physiological and pathological processes by regulating metabolic signals within the host’s body but also impact the intestinal immune system through various mechanisms while co-evolving with the mucosal immune system.^5^ Studies have indicated that disruptions of the balance in intestinal flora structure and metabolism can lead to dysfunction in multiple organs, resulting in chronic diseases such as inflammatory bowel disease,^6^ obesity,^7^ cardiovascular disease,^8^ and diabetes^9^ (Figure 1). Although advancements in our understanding have been made, the precise mechanisms through which intestinal flora influence the onset of disease remain elusive and are now a major focus of research. Emerging literature indicates a strong association between alterations in intestinal flora and immune-related diseases (Table 1). With the deepening of research, it was found that many T1DM patients and animal models have an imbalance of intestinal microorganisms.^31–34^

The imbalance of intestinal flora will destroy the normal function of digestion, metabolism, immune and nervous system, and then lead to obesity, diabetes, inflammatory bowel disease, allergic disease and other problems. Maintaining a balanced intestinal flora is essential for overall health.

**Table 1.** Common diseases caused by dysbiosis of the intestinal flora.

Disease type	Changes of intestinal flora		Possible mechanism		Reference	
Pancreatitis	The number of probiotics decreased and the number of potential pathogens increased		Abnormal intestinal flora composition can promote the release of inflammatory mediators that travel through the blood circulation to the pancreas, triggering or exacerbating the inflammatory response		^10,11^	
Crohn’s disease	The number of anaerobic bacteria such as *Bacteroides* decreased, and the number of *Fusobacterium* increased		The imbalance of flora causes the immune system to overreact and induces intestinal inflammation		^6,12^	
Colorectal cancer	*Bifidobacteria* and *Lactobacillus* decreased, *Fusobacterium* and *Actinobacteria* increased		The imbalance of intestinal flora may lead to the impairment of the intestinal mucosal barrier, allowing pathogenic microorganisms and their metabolites to penetrate the intestinal wall into the mesenteric lymph nodes and other lymphoid tissues, stimulating and exacerbating the inflammatory response		^13,14^	
Gastric cancer	*Lactobacilli* decreased, *Bacteroides* and *Clostridia* increased		The imbalance of intestinal flora can promote the occurrence of gastric cancer by inducing inflammatory response		^15,16^	
Liver cancer	The beneficial bacteria decreased and the harmful bacteria increased		The imbalance of intestinal flora destroys intestinal barrier function, increases intestinal permeability, causes bacteria and their metabolites to enter the portal vein system and reach the liver, inducing inflammation in the liver		^17,18^	
Obesity	*Prevotella* increased, *Bacteroides* decreased		Changes in microbiota affect energy budget and fat storage		^7,19^	
Diabetes	*Prevotella* decreased, *Bacteroides* increased		The imbalance of flora leads to autoimmune destruction of islet beta cells and induces diabetes		^8,20,21^	
Cardiovascular disease	anaerobic bacteria decreased and conditioned pathogens increased		Dysbiosis affects blood lipid metabolism, inflammation and increases the risk of cardiovascular disease		^9,22^	
ColorecAlzheimer’s disease	The number of probiotics decreased and the number of potential pathogens increased		The imbalance of intestinal flora leads to impaired intestinal barrier function, promotes the entry of harmful microorganisms and their metabolites into the blood circulation, and stimulates systemic and localized inflammatory responses. Inflammatory factors such as TNF-α and IL-1β cross the blood-brain barrier, affect the function and structure of brain neurons, and accelerate neurodegeneration		^23,24^	
Allergy	*Lactobacillus* and *Bifidobacterium* decreased		Dysbiosis affects immune system function and inflammatory response		^25,26^	
Pneumonia	*Klebsiella*, *Enterococcus*, *Escherichia coli* increased; *Lactobacillus* and *Bifidobacteria* decreased		The imbalance of gut microbes leads to impaired gut barrier function, promoting harmful substances in the gut to enter the blood circulation and reach the lungs, activating the lung inflammatory response, thereby increasing the risk of pneumonia		^27,28^	
Nephritis	The number of probiotics decreased and the number of potential pathogens increased		An imbalance of gut microbes leads to abnormal activation of the immune system, triggering inflammation in the kidneys		^29,30^	

The composition of intestinal flora in animal models and T1DM patients differs significantly from that of healthy individuals, primarily in two aspects. Firstly, there is a reduction in the diversity of intestinal flora in T1DM. Secondly, there is a notable increase in *Bacteroidetes* and decrease in *Firmicutes* within the T1DM group, along with an elevated presence of potentially pathogenic bacteria such as *Proteobacteria*. Further researches have revealed that complex interactions between intestinal flora, permeability, and the immune system contribute to the pathophysiological changes associated with T1DM.^35,36^ This review aims to elucidate the complex interactions between intestinal flora structure and its metabolites with T1DM development, providing new insights for effective prevention and treatment strategies.

## Involvement of intestinal flora in the possible pathogenesis of T1DM

**Figure 2.**
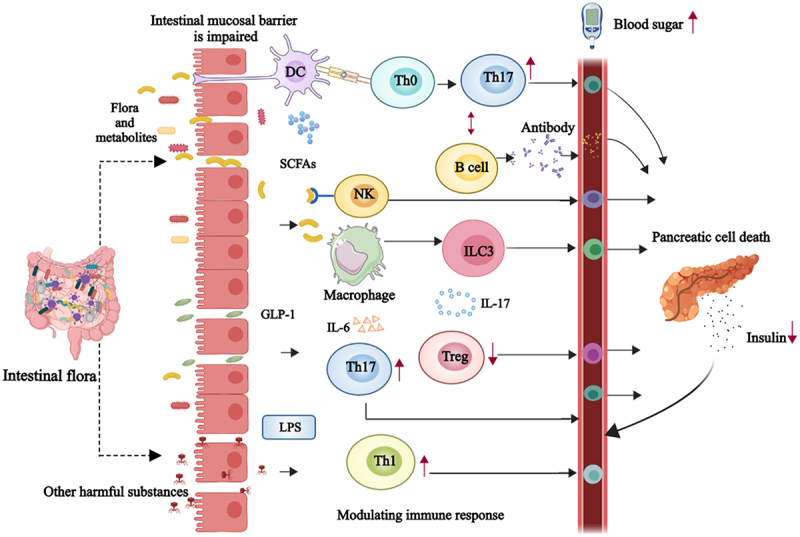
Intestinal flora and metabolites in the pathogenesis of T1DM.

Intestinal flora, particularly in human metabolism and immune regulation, plays an important role in the pathogenesis of T1DM. Although the relationship between intestinal flora and T1DM is not yet fully understood, increasing evidence has shown that intestinal flora and its metabolites can influence various physiological processes, such as intestinal mucosal barrier function, insulin secretion, and regulation of the local and systemic immune system. These effects may contribute to autoimmune attack and destruction of pancreatic islet β-cells, thereby leading to the development of T1DM (Figure 2).

The intestinal flora and its metabolites compromise the integrity of the intestinal mucosal barrier, leading to increased intestinal permeability. This increased permeability facilitates the entry of antigenic substances such as LPS into the bloodstream, triggering T cell activation and the release of pro-inflammatory cytokines like IL-6 and IL-17. This sequence of events initiates an inflammatory response that targets and destroys islet β-cells, thereby precipitating the onset of T1DM. Abbreviations: DC, Dendritic cells; B cells, B lymphocyte; Th0, Th0 cell; Th1, Th1 cells; Th17, Th17 cells; NK, natural killer cells; ILC3, Group 3 innate lymphoid cells; Treg, Regulatory T cell; IL-6, Interleukin-6; IL-17, Interleukin 17; GLP-1, glucagon-like peptide-1; LPS, Lipopolysaccharide; SCFAs, Short-chain fatty acids.

### Intestinal flora are involved in the pathogenesis of T1DM by altering the intestinal mucosal barrier

The intestinal mucosal barrier consists of intestinal epithelial cells, a mucous layer, tight junction proteins, and immune cells, serving as the intermediary link between intestinal flora and the host.^37^ In addition to its role in digestion and absorption, a fully functional mucosal barrier also prevents harmful substances in the gut (such as pathogenic bacteria and toxins) from entering other tissues, organs, and the bloodstream through the intestinal mucosa.^38^ Persistent inflammation or infection of intestinal tissues can damage the intestinal mucosal barrier, allowing bacterial translocation.^39^ In the early stages of T1DM, an imbalance in intestinal flora can impair intestinal barrier function, enabling pathogenic bacteria and their metabolites to breach the intestinal mucosal barrier and enter the bloodstream, triggering chronic inflammation in the pancreas.^40^ In the later stage of T1DM, loss of islet β-cells function causes large amounts of metabolic waste to enter the intestinal lumen, altering the environment and exacerbating the imbalance of intestinal flora.^41^ T1DM and intestinal flora imbalance interact and regulate each other, perpetuating a vicious cycle.

MIRANDA et al.^42^ observed significant impairment of intestinal mucosal barrier function in non-obese diabetic (NOD) mice and streptozotocin (STZ)-induced T1DM mice in their studies. In that study, they also detected translocated intestinal flora in the pancreatic lymph nodes of T1DM mice. Subsequent investigations have demonstrated that these translocated bacteria can induce the differentiation of CD4^+^ T cells into Th1 and Th17 subtypes, leading to local inflammatory responses and increased intestinal permeability in T1DM. This occurs through the activation of nucleotide-binding oligomerization domain 2 receptors on CD11b^+^ myeloid cells within the pancreatic lymph nodes. Additionally, Bosi et al.^43^ assessed intestinal mucosal permeability using a lactulose-mannose penetration test and similarly found significantly elevated intestinal mucosal permeability in T1DM patients compared to the general population.

Lipopolysaccharide (LPS) in the outer membrane of gram-negative bacteria is considered a biomarker for increased intestinal mucosal permeability. When there is an imbalance in intestinal flora and damage to the intestinal barrier, intestinal mucosal and capillary permeability increase.^44^ This allows a significant amount of LPS to enter the bloodstream through the damaged intestinal mucosa, resulting in a marked elevation of LPS levels in peripheral blood.^45^ A recent study has shown that intestinal flora can affect the integrity of the intestinal mucosal barrier, leading to the infiltration of bacteria and toxins in the gut, which in turn activates the immune system, triggering an inflammatory response and the production of inflammatory factors such as tumor necrosis factor α (TNF-α) and IL-17.^46^ These inflammatory factors may attack the islet β-cells, ultimately leading to the development of T1DM. Sofi et al.^47^ discovered that serum LPS and endotoxin levels were significantly higher in T1DM patients than healthy controls. QIN et al.^48^ observed that STZ-induced C57BL/6J T1DM mice exhibited significantly decreased expression of colonic tissue mucosal epithelial tight junction proteins zonula occludens-1 and occludin compared to normal mice. Additionally, there was a notable increase in the expression of inflammatory cytokines interleukin 1β (IL-1β), interleukin 6 (IL-6), and TNF-α. These studies demonstrate that T1DM leads to impaired intestinal mucosal barrier function, which is mainly characterized by increased intestinal mucosal permeability.

Short-chain fatty acids (SCFAs) are bacterial metabolites produced through the metabolism of carbohydrates by intestinal bacteria, mainly consisting of acetic acid, propionic acid, and butyric acid. They are generated by *Clostridium*, *Bacteroides*, and *Bifidobacterium*, and play a role in the proliferation and differentiation of intestinal epithelial cells, as well as in reducing apoptosis and enhancing intestinal barrier function.^49^ Researchers used gas chromatography-mass spectrometry to measure the concentration of SCFAs in the feces and serum of T1DM patients, finding significantly lower levels of acetic, propionic, and butyric acids compared to controls. They hypothesized that this result might be due to a decrease in the number of SCFA-producing bacteria, failing to effectively protect the physical barriers of the intestine.^50^ Additionally, intestinal flora can participate in the metabolism of 4%-6% tryptophan, and its metabolites can act as ligands for the aromatic hydrocarbon receptor (AHR). AHR is widely expressed in intestinal epithelial cells and immune cells. Activation of the AHR signaling pathway induces the differentiation of type 3 innate lymphoid cells (ILC3) expressing the nuclear receptor retinoic acid receptor-related orphan receptor γt, which stimulates ILC3 to secrete interleukin 22. This, in turn, protects the colonic crypts from invasion by *Clostridium Citrobacter*.^51,52^ Therefore, we speculate that the disturbed intestinal flora in T1DM can also affect the intestinal mucosal barrier through its metabolites.

In conclusion, the intestinal flora and its metabolites impact the immune activation by regulating the integrity and permeability of the intestinal mucosal barrier, playing a crucial role in the onset and progression of T1DM. Despite some advancements in understanding how intestinal flora and its metabolites influence T1DM through the intestinal mucosal barrier, there are still numerous limitations and challenges. Specifically, there is a lack of understanding regarding the cellular and molecular mechanisms through which intestinal flora specifically affects T1DM via the intestinal mucosal barrier. Additionally, deficiencies exist in various aspects including technical methodologies, ethical considerations, and regulatory issues. Further research is needed to explore the specific mechanisms through which microbiota and metabolites impact intestinal epithelial cells, immune cells, and tight junction proteins such as occludin and claudin. This will enhance our comprehension of the intestinal flora’s role in the development of T1DM and facilitate the development of more effective treatment strategies.

### Intestinal flora is involved in T1DM by influencing insulin secretion

Mounting evidence indicates that the intestinal flora can directly or indirectly impact insulin secretion and metabolism through multiple mechanisms, which are pivotal in the onset and progression of T1DM. Firstly, intestinal flora can modulate insulin secretion by influencing gut hormone release.^53^ Islet glucagon-like peptide-1 (GLP-1) and gastric inhibitory peptide are secreted by intestinal L and K cells, rapidly released postprandially, and stimulate insulin secretion. Moreover, GLP-1 promotes insulin secretion and protects pancreatic β-cells by reducing apoptosis and promoting regeneration.^54^ Martchenko et al.^55^ demonstrated that intestinal L cells secrete intestinal glucagon and GLP-1, contributing to the maintenance of a 24-hour circadian rhythm of metabolic homeostasis. Furthermore, correlation analysis between the primary L cells transcriptome and the gut microbiome has revealed a certain degree of dependence of GLP-1 regulation on microbiota. Secondly, the gut-brain axis is a complex signaling network where intestinal flora regulates insulin secretion through interactions involving the nervous, hormonal, and immune systems.^56,57^ The vagus nerve serves as an important bridge within this axis^58^; studies have shown that SCFAs can stimulate insulin secretion via activation of the vagus nerve.^59^ The intestinal flora and its metabolites can also influence the excitability of enteric neurons, impacting the function of the gut-pancreatic axis. For example, intestinal flora can promote the production of SCFAs like isopropionic acid and butyric acid by influencing the concentrations of GLP-1 and insulin-like growth factor-1. These metabolites can stimulate intestinal neurons and enhance insulin secretion through neuro-endocrine reflexes.^60^

It is well known that sugar, lipid, and protein metabolism are interrelated and constrained by each other. When the metabolism of one substance is impaired, it can cause metabolic disorders in others and lead to corresponding diseases. Studies have shown a significant relationship between insulin sensitivity and either increased cholesterol synthesis or decreased cholesterol absorption. With the progress of research, it has been found that some intestinal flora can affect cholesterol metabolism and thus insulin sensitivity. Hu et al.^61^ found that the enrichment of intestinal *Desulfovibrionales* bacteria contributed to increase intestinal cholesterol absorption. However, increased levels of the metabolite hydrogen sulfide induce hepatic farnesoid X receptor expression and inhibit CYP7A1 protein expression, which enhances the cholesterol transporter proteins ATP-binding cholesterol transporter proteins Abcg5/g8 and promotes elevated cholesterol secretion in bile. This disruption in metabolic balance leads to insulin resistance and hyperglycemia. Recent research using multi-omics technology has found that intestinal flora can directly act on pancreatic β-cells through the production of metabolites such as tyrosine, stimulating insulin secretion.^62^ Notably, pyruvate and butyric acid can also reduce blood glucose levels and increase insulin sensitivity by regulating glycogen synthesis and gluconeogenesis in hepatocytes, which helps alleviate the symptoms of T1DM patients.^63^

With the advancement of research, it has been discovered that intestinal bacteria such as *Bifidobacterium*, *Lactobacillus*, and *Bacteroides* can produce metabolites like soluble cellulose, propionic acid, and lactic acid. These metabolites not only stimulate insulin secretion and lower blood sugar levels but also modulate immune system responses, protect pancreatic β-cells from autoimmune attacks, and reduce the risk of autoimmune reactions.^64^ In addition, an analysis of the composition and function of the gut microbiome in children with T1DM showed that the abundance of *Ruminococci* and *Anaplasmodial bacilli* was increased, while the abundance of *Bifidobacteria* and *Faecal bacilli* was decreased. Increased intestinal permeability, along with elevated levels of serum pro-inflammatory cytokines (e.g., IL-1β, IL-6, and TNF-α) and LPS, were also observed in T1DM.^65^ It is further suggested that intestinal flora can impact the pathogenesis of T1DM through the production of endotoxins and inflammatory cytokines, which can impair insulin secretion and metabolism.

In conclusion, a healthy intestinal flora can prevent or delay the onset of T1DM. An imbalance in intestinal flora can reduce the production of SCFAs, decrease the secretion of GLP-1, and increase inflammation, leading to decreased insulin secretion and the destruction of islet β-cells, thereby increasing the risk of T1DM. Although the intestinal flora plays an important role in T1DM by regulating insulin secretion, the complexity of its mechanisms and individual differences in intestinal flora make consistent results difficult to achieve, requiring further in-depth research. In addition to these limitations, future research should focus on explore new directions. A promising strategy is strengthening interdisciplinary cooperation among microbiology, immunology, and metabolomics fields. This approach aims to provide more comprehensive and effective theoretical support for the prevention and treatment of T1DM.

### Intestinal flora modulates T1DM by influencing immune modulation

T1DM is an autoimmune disease characterized by the immune system attacking islet β-cells, leading to inadequate insulin secretion and hyperglycemia. While the precise mechanism of islet β-cells destruction remains unknown, evidence suggests autoantibodies against these cells in T1DM, involving various innate and adaptive immune cells in the autoimmune response.^66^ Recent research indicates that intestinal flora and its metabolites play a significant role in the onset and progression of T1DM through their influence on immune regulation, involving modulation of diverse immune cells and signaling pathways.^67^

Closely related to intestinal flora and intestinal immune system and the intestinal tract play a crucial role in the immune system. The intestinal flora regulates immune cells and molecules in the intestine to maintain immune balance.^68^ Research has shown that intestinal flora and its metabolites can affect the types and quantities of various immune cells in the gut, including regulatory T cells (Tregs), effector T cells, natural killer cells (NK), dendritic cells (DCs), and macrophages.^69,70^ These cells are significantly involved in the development of T1DM, particularly in regulating autoimmune responses and inflammatory processes.

A previous study of a T1DM mouse model^71^ found that autoantibodies directed only against β-cells were not able to induce pancreatic β-cells destruction; whereas, T cells infiltration in pancreatic sections was suggested that β-cells specific autoreactive T cells play a key role in T1DM pathogenesis. NOD mice spontaneously develop T1DM; however, they do not develop pancreatic islet inflammation if they lack MHC class I molecules to present antigens to CD8^+^ T cells.^72^ Additionally, depletion of CD4^+^ T cells in NOD mice inhibits the development of T1DM.^73^ Studies have shown that intestinal flora can induce the proliferation of specific types of T cells, thereby influencing the immune response. For example, *Akkermansia* and *Mycobacterium anisopliae* can induce the proliferation of Th17 cells,^73^ and the overactivation of Th17 cells is thought to be a key factor in the development of T1DM.^74^ Furthermore, intestinal flora can also influence the differentiation and function of Treg cells, an important class of immunosuppressive cells capable of suppressing autoimmune responses. One study found that commensal bacteria can promote the differentiation and function of Treg cells, thereby suppressing autoimmune responses and slowing the development of T1DM.^75^ It has also been shown that inoculation of SCFA in NOD mice increases the number of Treg cells in the pancreas, achieving the same effect.^76^ It was also found that *Lactobacillus* and other probiotics can promote the secretion of IL-10 and other anti-inflammatory cytokines by DCs, reduce the production of IL-6, IL-12, IL-1β, and other pro-inflammatory cytokines, thereby promoting the generation of Tregs and inhibiting autoimmune reactions.^77,78^

Further investigation discovered that metabolites produced by intestinal flora can also modulate immune responses by influencing the metabolism and function of immune cells. Studies suggest that bacterial cathelin-related antimicrobial peptide (CRAMP) protects pancreatic islet β-cells from damage by inhibiting the induction of inflammatory factors in macrophages and DCs.^79,80^ Zhao et al.^81^ found that butyrate induced the expression of CRAMP via the SCFA receptors GPR41 and GPR43, and these receptors were also detected in pancreatic islet β-cells. Interestingly, when the mice were given antibiotics to eliminate the gut bacteria, CRAMP expression in the pancreas decreased. This finding further suggests that the development of T1DM is closely related to intestinal flora. It was also found that algal polysaccharides secreted by worms increase the number of *rumenococci* in the gut, which in turn inhibits the development of STZ-induced T1DM mice by inducing CD8^+^ Treg cells.^82^ Consistently, the number of *rumenococci* in the feces and the number of CD8^+^ Treg cells in peripheral blood mononuclear cells were positively correlated in T1DM patients, and both were lower than in normal controls.^83^

Recent research has revealed that the intestinal flora can modulate the activation and inhibition of various immune signaling pathways through interacting with intestinal epithelial cells and immune cells. These pathways include toll-like receptors (TLRs, such as TLR2 and TLR4),^84^ the nuclear factor-κB signaling pathway,^85^ the RIG-I receptor signaling pathway,^86^ and others. These pathways are pivotal in regulating the inflammatory response of immune cells and the onset of autoimmune diseases. Furthermore, the intestinal flora affects not only the intestinal immune system but also the development of T1DM through systemic immune regulation. Alterations in the intestinal flora can influence the immune response of distant organs and tissues, such as the pancreas. This occurs via inflammatory responses within the intestinal mucosa and the migration/activation of immune cells.^87^ It is noteworthy that an imbalance in intestinal flora may compromise intestinal barrier function, leading to the translocation of endotoxins like LPS and other harmful substances into the circulation through the intestinal mucosa, triggering a systemic inflammatory response.^88^ This chronic low-grade inflammation may play a crucial role in regulating immunity related to T1DM and causing damage to islet β-cells.

In conclusion, the intestinal flora and its metabolites play a crucial role in regulating the immune response by modulating T cells subsets, influencing inflammatory and autoimmune responses, and reducing the autoimmune attack on islet β-cells. This has significant implications for the onset and progression of T1DM. However, the specific roles and interactions of these mechanisms are still not fully understood. Further investigation into how intestinal flora and its metabolites interact with different immune cell types and signaling pathways is necessary to elucidate their precise involvement in T1DM development and to establish a scientific foundation for personalized treatment strategies in the future.

## Targeting intestinal flora and their metabolites to improve T1DM

**Figure 3.**
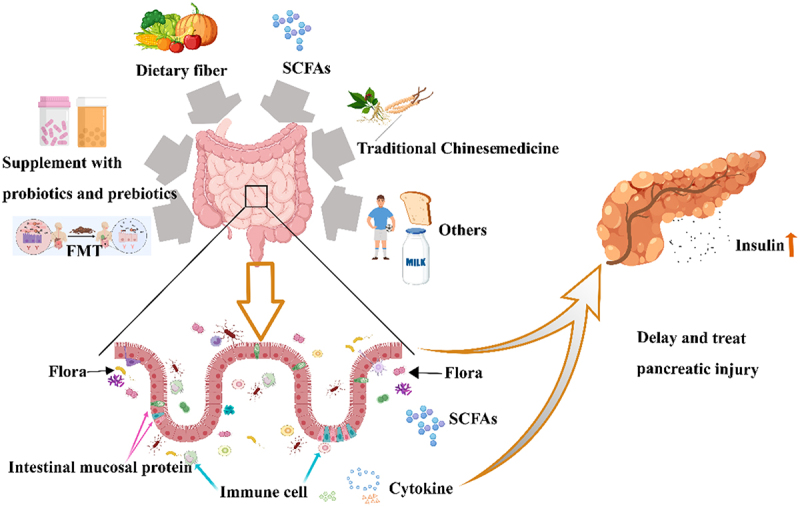
Multiple interventions have the potential to prevent and treat T1DM by regulating the intestinal flora and its metabolites.

The intestinal tract harbors a diverse array of probiotic and pathogenic bacteria. Intestinal flora and their metabolites play crucial roles in regulating immune function, energy metabolism, and hormone levels. Currently, there is a global effort to treat various diseases by targeting specific bacteria, and some of these relevant bacterial groups have registered their clinical trials and treatment results with the World Health Organization (Table 2). Due to the imbalance of intestinal flora, pathogenic bacteria dominate, abnormally activate the immune system, trigger autoimmune reactions, and ultimately lead to reduced insulin secretion and the development of T1DM. Therefore, intervention in intestinal flora may potentially prevent and treat T1DM (Figure 3).

The intestinal flora and its metabolites can be effectively modulated by fecal microbiota transplantation, probiotics, dietary fiber, short-chain fatty acids, traditional Chinese medicine, and other interventions. These methods directly enhance the integrity of the intestinal mucosal barrier and indirectly modulate the immune system, ultimately aiding in the prevention or management of T1DM. Abbreviations: FMT, fecal microbiota transplantation; SCFAs, Short-chain fatty acids.

**Table 2.** A wide range of potential applications of specific intestinal flora in medical treatment.

Target bacteria	Source	Identifier	Study disease	Dosage form	Status
*Bifidobacterium*	ClinicalTrials.gov	NCT06325605	Adolescent Depression	Capsule	Not Applicable
	ICH GCP	NCT04982380	Type 2 Diabetes Mellitus With Complication	Tablet	Phase 4
	ICH GCP	NCT05620004	Advanced Hepatocellular Carcinoma	Oral liquid	Phase 1/2
	ICH GCP	NCT05662514	Helicobacter Pylori Infection	Granules	Not Applicable
	ClinicalTrials.gov	NCT03880760	Type 1 Diabetes Mellitus	Capsule	Not Applicable
*Lactobacillus reuteri*	ClinicalTrials.gov	NCT01587846	Abdominal Pain and Constipation – Functional	Tablet	Phase 3
	ICH GCP	NCT04577625	Healthy	Capsule	Not Applicable
	ICH GCP	NCT04262648	Infantile ColicConstipation; Infantile; DiarrheaGastro; Esophageal Reflux	Drops	Not Applicable
*Lactobacillus Gasseri* BNR17	ICH GCP	NCT04260997	Weight Management	Capsule	Not Applicable
*Lactobacillus* brevis CD2 lozenge	ClinicalTrials.gov	NCT01545687	Head and Neck Cancer; Oral Complications of Radiation Therapy; Pain; Weight Changes	Tablet	Phase 3
*Lactobacillus*	ClinicalTrials.gov	NCT05974449	Breast Cancer Female	Capsule	Phase 2
*Bacteroides Thetaiotaomicron*	ClinicalTrials.gov	NCT02704728	Crohn’s Disease	Capsule	Not Applicable

### Intestinal flora transplantation

Intestinal microbiota transplantation (IMT), also known as fecal microbiota transplantation (FMT), involves transplanting the gut microbiota from a healthy individual into a patient’s gut to improve their intestinal microecological balance. It has been suggested as a potential therapeutic approach to treat T1DM by modifying the intestinal flora. Based on the results of existing studies, IMT affects T1DM in three main ways: first, as an intervention method to prevent or delay the onset of T1DM; second, to prevent the development of high-risk clinical symptoms in T1DM patients; and third, to alleviate clinical symptoms in T1DM patients who already exhibit them.

It has been demonstrated in animal models that transplantation of a healthy microbiota can prevent or delay the onset of T1DM by enhancing regulatory T cell function and reducing pro-inflammatory cytokines.^89^ Other animal experiments also suggest the potential protective effect of intestinal flora transplantation on T1DM. For instance, experiments with intestinal flora transplantation in both wild and laboratory mice demonstrated a reduction in T cell-mediated autoimmune responses and a lower incidence of T1DM when healthy intestinal flora were transplanted.^90^ Furthermore, it has been shown^91^ that transplantation of intestinal flora from NOD mice lacking the toll-like receptor junction molecule myeloid differentiation factor 88 resulted in increased secretion and expression of immunoglobulin A (IgA) and TGF-β in the intestinal tract. This improvement in the damaged intestinal mucosal barrier and its function helped prevent and delay the development of T1DM in NOD mice. Several small clinical trials have also reported promising results with IMT for treating T1DM, including enhanced glycemic control, reduced insulin requirements, and increased levels of SCFAs in the gut.^92,93^ One study indicated that patients with T1DM treated with FMT experienced reduced insulin use and lower fasting blood glucose (FBG) levels over time.^94^ Furthermore, IMT improved the diversity and stability of patients’ intestinal flora.^95^

Despite the potential benefits of FMT for T1DM, there are still many challenges and uncertainties that need to be addressed, including donor selection, standardization of fecal preparation, and safety and efficacy for long-term use. The variation in intestinal flora composition among individuals represents a significant hurdle for successful FMT, as compatibility between donor and recipient must be carefully considered.^96^ Mismatched flora transplantation may disrupt the recipient’s gut ecology and cause new health issues. Furthermore, dynamic changes in intestinal flora due to environmental factors, diet, and medication can lead to instability in therapeutic effects even after a successful transplant.^97^ Donor selection, fecal preparation, and long-term safety and efficacy are all areas of concern that require further research to establish strict clinical and regulatory standards.

### Probiotic and prebiotic supplementation

Probiotics are beneficial microorganisms such as *Lactobacilli* and *Bifidobacteria*. When administered in appropriate amounts, they can improve the body’s immune function and reduce the risk of autoimmune reactions by balancing the intestinal microecosystem. Probiotics can also promote the integrity of the intestinal mucosal barrier and enhance the immune function of intestinal epithelial cells.^98^ Increasing prebiotic levels can enhance intestinal mucus secretion, improve intestinal motility, and decrease the number of harmful bacteria in the gut.^99^

Research has shown that changes in the intestinal flora after taking certain amounts of probiotics can increase the proportion of SCFA-producing bacteria, which may have a positive effect on delaying the development of T1DM.^79^ For example, Hansen et al.^100^ found that administering prebiotic xylooligosaccharides to mother and offspring mice delayed the onset of diabetes and reduced insulin dependence. This treatment also led to less infiltrating inflammatory cells in pancreatic islets and salivary glands, decreased autoimmune responses, and lower intestinal permeability markers in both the small and large intestines. In addition, it will also lead to a stronger anti-inflammatory environment both locally and systemically, which is mainly by inhibiting the expression of proinflammatory cytokines and increased levels of anti-inflammatory cytokines, as well as higher abundance of activated Tregs.^101^ Short-term feeding of probiotic galacto-oligosaccharides (GOS) increased the abundance of specific saccharolytic bacteria (species of Bacteroides and Lactobacillus), increased the abundance of β-galactosidases in young and old animals, and increased the non-saccharolytic organisms; and elevated IL-17 and IL-6 levels in serum.^102^ Wang et al.^103^ demonstrated that a GOS diet could lead to some improvement in microbiome dysbiosis, inflammation and gut barrier defects. They found that probiotics can improve gut barrier function by increasing the levels of SCFAs-producing bacteria and SCFAs, enhancing claudin-1 and mucin-2 expression, and decreasing the levels of *Escherichia coli* and LPS. Moreover, probiotics enhance insulin secretion by up-regulating G protein-coupled receptor (GPCRs) 43/41, proglucagon, and proconvertase 1/3 activity, which triggers glucose-induced GLP-1 secretion and reduces pancreatic β-cells apoptosis to some extent.^103^ A clinical systematic evaluation and meta-analysis^104^ found that probiotic and prebiotic supplementation improved FBG, hemoglobin A1c (HbA1c), and C-peptide levels in patients with T1DM, while reducing insulin requirements to a certain extent. Additionally, researchers have also found that diabetic patients supplemented with probiotic yogurt have lower FBG and HbA1c levels, along with increased in erythrocyte superoxide dismutase, glutathione peroxidase activity, and total antioxidants.^105^

In summary, modulation of intestinal flora using probiotics and/or prebiotics can be a dietary intervention for the prevention and treatment of T1DM. Nevertheless, its efficacy and safety are confronted with numerous challenges and risks. Variations in individuals’ intestinal flora composition and immune system status may result in different responses to prebiotics and probiotics.^106^ The same prebiotics or probiotics may elicit diverse effects on different individuals, with some benefiting while others experiencing ineffectiveness or even adverse reactions.^107^ Given the complex immune mechanisms involved in T1DM, the impact of probiotics and prebiotics on the immune system remains incompletely understood. Prolonged use may induce adaptive changes in the immune system, potentially leading to tolerance or dependence, which could affect immune function within the gastrointestinal tract and throughout the body.^108^ Furthermore, there is a wide array of probiotic and prebiotic products available on the market, each varying in quality and ingredients, coupled with a lack of standardized regulations and oversight. Consequently, research is imperative to elucidate their mechanisms of action and long-term effects, while confirming their efficacy and safety through rigorous clinical trials. Simultaneously, enhancing product quality control measures and standardization efforts are essential to ensure stability and consistency across probiotic and prebiotic products, ultimately providing reliable treatment options for patients with T1DM.

### Dietary fiber supplementation

In addition to medication, dietary factors can also have an important role on the glycemic control of T1DM patients. Among them, dietary fiber, a key nutrient, has become one of the effective strategies to improve blood glucose in T1DM patients. Dietary fiber refers to carbohydrates that cannot be digested or absorbed by the body. It includes two types: soluble and insoluble fiber. In addition to slowing down food, digestion soluble fiber can also regulate blood lipids and blood pressure by adsorbing cholesterol, fatty acids, and other substances.^109,110^ Insoluble fiber, on the other hand, can increase fecal volume, softness and relieve constipation.

In a study by Liu et al.^111^ using alloxan-induced diabetic mouse model, dietary fiber was shown to control blood glucose, improve glucose tolerance, and reduce serum insulin levels in diabetic mice. It also reduced total cholesterol levels and the number of *Mycobacterium anisopliae*, *Ascomycetes*, and *Ruminalococci* families, while increasing the abundance of *Firmicutes*, *Lactobacillus*, and *Prevotellaceae*. Another study explored the effects of buckwheat bran soluble dietary fiber (SE-SDF) on mice. The findings revealed that SE-SDF enhanced oral glucose control by activating hepatic PI3K/Akt/FoxO1 and GPR43/AMPK signaling pathways. Additionally, it modulated the intestinal flora-SCFAs-GPR43/GLP-1 signal transduction axis, which improved tolerance, reduced insulin resistance, and decreased injuries in the liver, pancreas, and colon of diabetic mice.^112^ Further, one study found that dietary fiber can increase the type and number of SCFAs-producing flora, as well as promote the production of GLP-1, which may be a contributing factor to the improvement of HbA1c levels in patients.^113^ Subsequent analysis revealed that dietary fiber increases the number of *Bifidobacterium lactis*, which enhances glycogen synthesis, reduces the expression of hepatic gluconeogenesis genes, improves glucose transporter-4 translocation, and promotes glucose uptake.^113^ Recently, a new high-energy oral dietary supplement (ONS) has been developed for individuals with diabetes or prediabetes, containing slowly digestible isomaltulose and soluble dietary fiber. To evaluate the effect of ONS on blood glucose levels in pre-diabetic patients, researchers conducted a single-blind, randomized crossover clinical trial with 20 participants. Results showed that a low proportion of available carbohydrate (LC-ONS) (40% energy from carbohydrates) consistently maintained lower blood glucose levels compared to standard ONS (54% energy from carbohydrates). Additionally, the increase in the plasma insulin curve was significantly smaller following LC-ONS ingestion.^114^

In addition to the above studies, several other studies have shown that dietary fiber has a positive effect on health and glycemic control in patients with diabetes.^115^ These findings suggest that dietary fiber supplementation is a simple but effective way to improve glycemic control in T1DM patients. Therefore, it is recommended that T1DM patients moderately increase dietary fiber intake in their daily diets and choose foods rich in soluble fiber, such as oats, beans and apples. In addition, supplementing some high-fiber drinks, such as rye fiber drink and oatmeal drink, is also a good choice. It is worth noting that there is limited research on the long-term effects of dietary fiber in the treatment of T1DM, and it is the long-term benefits remain unclear. Future research should focus not only on individual differences, digestive side effects, nutritional balance issues, fiber type and quality, and long-term effects of dietary fiber but also explore the optimal type, dosage, and application. This will aid in establishing personalized treatment protocols that ensure their safety, effectiveness, and patient compliance.

### Supplementation of SCFAs

SCFAs, as one of the metabolites of intestinal flora, are produced by the fermentation of dietary fiber by intestinal flora, including propionic acid, butyric acid, and acetic acid, which are absorbed into the blood circulation through intestinal epithelial cells. It has been demonstrated that SCFAs, as specific ligands for two GPCRs, GPR43/FFA2 and GPR41/FFA3, influence host health at the cellular, tissue, and organ levels through mechanisms related to intestinal barrier function, glucose homeostasis, immunomodulation, and obesity.^116,117^ Over the past decades, studies have found that SCFAs directly modulate insulin secretion and tissue sensitivity, reduce inflammatory responses, and improve islet cell function by affecting the immune system. Using messenger RNA and immunohistochemistry (IHC) assays, researchers have found that the receptors for free fatty acids (FFAs) are widely expressed in the gastrointestinal tract, pancreas, and most β-cells.^118^ Moreover, deletion of the *FFA2* gene leads to impaired glucose tolerance and insulin secretion, and even decreased the expression of β-cells markers (mafA, Pdx1, NeuroD).^119^ Interestingly, the stimulation of pancreatic β-cells proliferation with FFA2 agonists 2-butyric acid (SCA15) and 2-propionic acid (SCA14) not only induces pancreatic β-cell proliferation, but also enhances insulin secretion through Gαq/PLC-mediated activation of inositol 1,4,5-triphosphate (IP_3_) and calcium (Ca^2+^).^119,120^ It has also been found that increased SCFAs in the cecal lumen of pregnant mice increases the expression of SCFAs and FFA2 in the pancreas, which may stimulate islet β cell proliferation and insulin secretion and compensate for insulin resistance.^121^

After pretreating human islet cells with 1 mm propionic acid for 24 hours in vitro, researchers observed a significant reduction in the expression of inflammatory factors and a certain degree of alleviation in palmitate-induced cell death.^122^ It has also been found that long-term use of three SCFAs, sodium acetate, sodium propionate, and sodium butyrate, significantly enhanced pancreatic β-cells function.^123^ It has been clearly demonstrated that SCFAs can protect β-cells from damage caused by FFAs and inflammatory cytokines. Furthermore, a study has provided compelling evidence that FFA2 agonists (Schiff periodic acid Schiff) can enhance insulin secretion through the activation of Gαq/PLC-mediated IP3 signaling and Ca^2+^ release.^119^ In recent years, the use of SCFAs in the treatment of T1DM has emerged as a potential novel therapeutic approach. Moreover, researchers have explored the application of SCFAs in T1DM patients to assess their potential positive impact. In a cohort study on environmental determinants of diabetes in young adults, SCFAs have been shown to exert a protective effect against early-onset T1DM.^124^ Additionally, it has been observed that increased intake of SCFAs has been observed to promote the body’s consumption of tryptophan metabolites and omega-3 fatty acids, thereby facilitating improved metabolic control to delay the progression of T1DM.^125^

Due to the limited number of clinical studies on SCFAs treatment for T1DM, especially the lack of large-scale, long-term randomized controlled trials, there is insufficient scientific evidence to fully support their efficacy and safety. This may result in unclear treatment effects or substandard expected outcomes. The specific mechanism of SCFAs in T1DM needs further verification, along with special attention to bioavailability, metabolic issues, side effects, and safety. Further research is necessary to clarify their mechanisms of action and long-term effects, as well as to verify their efficacy and safety through detailed clinical trials. It is also important to enhance product quality control and standardization to ensure the safety and efficacy of SCFAs supplements.

### Conditioning effects of traditional Chinese medicine

In the theory of traditional Chinese medicine (TCM), the spleen and stomach are considered the source of biochemistry, and their functions directly affect the overall health of the human body. The intestinal tract is the extension of the spleen and stomach, which is important for the regulation and maintenance of intestinal microorganisms.

In both animal models and patients, the intestinal immune function in T1DM is dysregulated, leading to disturbances in intestinal flora and damage to the intestinal mucosal barrier. TCM can regulate the balance of the intestinal immune system, increase intestinal mucosal IgA levels, and reduce the intestinal mucosal inflammatory response, thus protecting the intestinal mucosal barrier. For example, the commonly used TCM formula ‘Sijunzi Decoction’ strengthens the spleen and regulates central qi, improving the function of the spleen and stomach, promoting the balance of intestinal flora, and increasing gamma globulin levels. This enhances the body’s immunity and protects the intestinal mucosal barrier.^126^ Xu et al.^127^ showed that the antidiabetic effects of Gegen Qinlian Decoction (GOD) and Xiaofangshi alkaloids, such as reducing inflammation and lowering blood glucose. These benefits were due to their ability to modify the overall structure of the intestinal flora and enrich a number of butyrate-producing bacteria, including *Bacillus faecalis* and *Rose Algae*. Interestingly, it was found that the combination of GOD with metformin significantly increased the levels of the target genes cell cycle protein-dependent kinase 4 (Cdk4) and insulin receptor substrate (Irs1), while also somewhat reduced the mortality rate of pancreatic islet cells in STZ-induced diabetic rats.^128^ Additionally, other scholars have found that GOD also improves insulin resistance, stabilizes regulate glucose and lipid metabolism, and reduces oxidative stress in the body, thereby achieving the purpose of treating diabetes.^129^ In clinical trials, it has been demonstrated that TCM can modulate the composition and abundance of the intestinal flora. For example, a randomized, double-blind, placebo-controlled clinical trial^130^ revealed that diabetic patients who took GOD for 12 consecutive weeks experienced changes in the number and structure of their intestinal flora. This was manifested by an increase in the number of beneficial bacteria (such as *Bifidobacteria* and *Lactobacilli*) and a decrease in the number of harmful bacteria (such as *Escherichia coli* and *Actinobacteria*), leading to a significant reduction in FBG and glycated hemoglobin levels, and improvements in pancreatic islet β-cells function.

However, there are challenges and risks associated with using TCM for T1DM treatment. Firstly, its efficacy is often based on experience, animal studies, and small-scale clinical trials, rather than on large-scale randomized controlled trials. This lack of robust scientific evidence may lead to uncertain treatment outcomes and increased risks. Second, the complexity of TCM composition makes quality control difficult; different batches may vary significantly in composition and potency, leading to inconsistent efficacy or even toxic side effects. Finally, the long-term safety of TCM has not been fully studied, and some TCM may exhibit potential toxicity. Long-term use may lead to impairment of liver or kidney function, or other side effects. Further research is needed to ensure its safety. Therefore, it is imperative to conduct large-scale and rigorously designed randomized controlled trials to validate the efficacy and safety of TCM in treating T1DM. Strong scientific evidence is essential to promote the standardized application of TCM in T1DM treatment. Furthermore, enhancing the quality control and standardization of Chinese medicinal materials is necessary to ensure consistent composition and efficacy across different batches. Standardization will improve the reliability and repeatability of TCM treatments. Only with sufficient scientific evidence and strict quality control can TCM provide safe and effective treatment options for patients with T1DM.

## Summary and outlook

The intestinal flora plays a critical role in the pathogenesis of T1DM. In this paper, the key role of intestinal flora and its metabolites in T1DM is reviewed in depth, and the latest findings on how intestinal flora and their metabolites affect T1DM are illustrated. In addition, by discussing potential therapeutic strategies for T1DM, including FMT, probiotic supplementation, dietary fiber intake, SCFAs supplementation, and TCM conditioning, this paper lays a foundation for future detailed exploration of potential interventions for T1DM. Furthermore, studies have suggested that dietary factors such as milk protein and gluten may contribute to the pathogenesis of T1DM by influencing the immune system or intestinal permeability. For instance, during digestion, casein A1 produces the peptide β-casein, which may trigger an immune response and increase the risk of T1DM.^131,132^ There is also evidence indicating a higher incidence of T1DM in individuals with celiac disease,^133,134^ possibly due to gluten-induced increased intestinal permeability leading to heightened antigen entry into the bloodstream, triggering an autoimmune response in susceptible individuals. However, due to substantial individual variations, there remains some controversy surrounding these findings. Ongoing research aims to further elucidate the specific effects and mechanisms of these dietary factors on T1DM.

While our understanding of the relationship between intestinal flora and T1DM is not yet complete, mounting evidence suggests that maintaining a healthy intestinal flora may hold potential for preventing and treating T1DM. So, the intricate interplay between intestinal flora and T1DM warrants further investigation. In a word, regulation of intestinal flora and its metabolites, as well as the identification of specific strains and metabolites, will offer novel insights into diagnosing and treating T1DM with promising prospects for future clinical interventions.
